# The Electronic Property Differences between dA::dG and dA::dG^oxo^. A Theoretical Approach

**DOI:** 10.3390/molecules25173828

**Published:** 2020-08-23

**Authors:** Boleslaw T. Karwowski

**Affiliations:** DNA Damage Laboratory of Food Science Department, Faculty of Pharmacy, Medical University of Lodz, ul. Muszynskiego 1, 90-151 Lodz, Poland; Boleslaw.Karwowski@umed.lodz.pl

**Keywords:** electronic properties, MutY, dA::dG and dA:::dG^oxo^ mismatch, 7,8-dihydro-8-oxo-2′-deoxyguanosine, DNA repair

## Abstract

The dA::dG^oxo^ pair appearing in nucleic ds-DNA can lead to a mutation in the genetic information. Depending on the dG^oxo^ source, an AT→GC and GC→AC transversion might be observed. As a result, glycosylases are developed during the evolution, i.e., OGG1 and MutY. While the former effectively removes G^oxo^ from the genome, the second one removes adenine from the dA::dG^oxo^ and dA:dG pair. However, dA::dG^oxo^ is recognized by MutY as ~6–10 times faster than dA:dG. In this article, the structural and electronic properties of simple nucleoside pairs dA:dG, dC:::dG^oxo^, dC:::dG, dA::dG^oxo^ in the aqueous phase have been taken into theoretical consideration. The influence of solvent relaxation on the above is also discussed. It can be concluded that the dA::dG^oxo^ nucleoside pair shows a lower ionization potential and higher electron affinity than the dA:dG pair in both a vertical and adiabatic mode. Therefore, it could be predicted, under electronic properties, that the electron ejected, for instance by a MutY 4[Fe-S]^2+^ cluster, is predisposed to trapping by the ds-DNA part containing the dA::dG^oxo^ pair rather than by dA::dG.

## 1. Introduction

**Figure 1:**
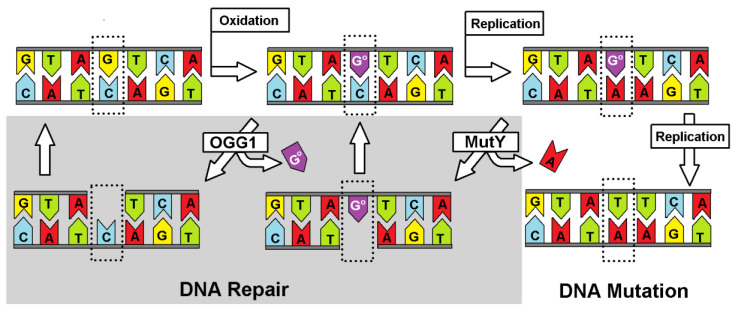
The repair pathway of the A::G^oxo^ base pair. G^oxo^, depicted as G^o^.

Genetic information, which is stored in the nucleobase sequence, is continuously exposed to harmful and exogenous factors such as reactive oxygen/nitrogen species, ionization radiation, pollution, etc. [1]. Their interaction with the genome gives rise to various types of DNA lesions. It is generally recognized that during one hour in the human body, which contains approximately 1 × 10^13^–1 × 10^14^ cells, 3 × 10^17^ DNA damage events can take place [2]. One of the most common is 8-oxo-7,8-dihydro-2′-deoxyguanosie (dG^oxo^), whose cellular level in a cell has been estimated as ~10^5^ per cell/per day, depending on the tissue type, exposure to different external factors, age, etc. [1]. Due to the mutagenic potential of different nucleic acid lesions, of which over 70 types are known, cells have developed several repair systems over the course of evolution [3]. Our basic understanding of them has been the subject of many review articles [4,5]. The most common and effective repair system, recognized as the line of first defense, is BER (Base Excision Repair), which is initiated by specific glycosylases [6]. It is important to mention here that more complicated lesions like inter- and intra-strand crosslinks, pyrimidine dimers, and 5′,8-cyclo-2′-deoxypurines among others are repaired by much more complicated systems such as nucleotide excision repair, homolog recombination, and non-homolog end-joining [7]. In the case of BER, glycosylases can recognize a modified nucleobase in the oligonucleotide structure and A) incise the glycosidic bond yielding an apurinic/apyrimidinic site as a substrate for an endonuclease and modified base or B) by a second activity cleave a 3′/5′-end phosphodiester bond of the formed AP-site leaving after a β/δ-elimination single-strand break (SSB) [8]. The lack or loss of the above protein activity can lead to a different kind of mutation and, therefore, can induce carcinogenesis [9]. On the other hand, glycosylases are the target enzymes for chemo or chemo/radiotherapy [10]. Their inactivation can switch the cancer cell from proliferation to an apoptosis or necrosis path [11]. Due to the susceptibility of dG to the one-electron oxidation process, glycosylases, which selectively recognize/remove dG^oxo^ and its further degradation product, are the focal point of scientific interest. As shown in Figure 1, the appearance of dG^oxo^ in the genome structure can lead to GC→AT transversion [12]. Additionally, because the dG^oxo^TP (dG^oxo^ triphosphate) is present in the cellular dNTP (2′-deoxynucleoside triphosphate) pool, and is a suitable substrate for polymerases, an AT→GC mutation can be observed, too [13]. To avoid these undesirable events and to keep genetic material reproducible and stable, over the course of evolution, cells developed several specific glycosylases, such as OGG1 (8-Oxo-Guanine Glycosylase 1), MutY (adenine DNA glycosylase), UDG (Uracil-DNA Glycosylase), etc. [14]. It is important to mention that the number of these protein copies in a cell is rather low and non-equal. MutY glycosylase exists in 30 copies per *Escherichia coli* (*E.coli*), in which several kilos of base pairs (BP) are under verification (5 × 10^5^ BP) [15,16].

Recently, it has been proposed that MutY, which is contained in a structure [4Fe-4S]^2+^ cluster, can effectively scan the genome by electron transfer between two “red-ox” proteins [17]. However, the iron-sulfur cluster is not required for glycosidic bond hydrolysis of 2′-deoxyadenosine by MutY. Otherwise, scanning the whole genome by a low number of glycosylase copies in a reasonable time is impossible if genetic information is to be kept free of errors. It should be pointed out that dA::dG^oxo^ is recognized by MutY as ~6–10 times faster than dA:dG [18,19]. For details, please see Barton and Wallace’s review article [20,21,22]. However, the proposed mechanism is preferred/suitable in the case when dG^oxo^ exists in a one-electron oxidizing state (radical cation), which is rather unstable in character and undergoes further rearmaments [22]. The time scale of the charge recombination process is around 5 × 10^3^ s^−1^ [23]. In this article, for the first time, the differences in electronic properties between the dA::dG^oxo^ and dA:dG pair have been given theoretical consideration.

## 2. Results and Discussion

### 2.1. Nucleoside Pair (NP) Structure and Its Electronic Properties

**Figure 2:**
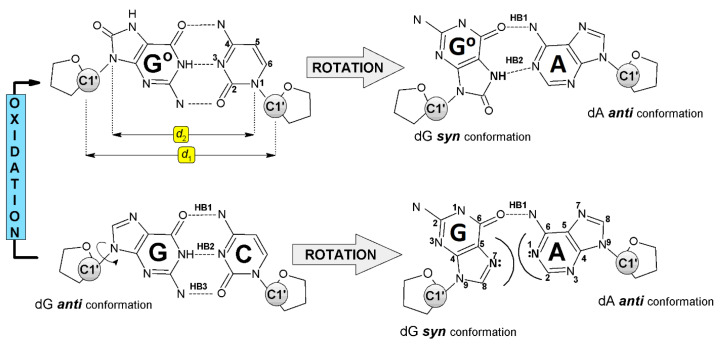
Structures of investigated nucleoside pairs with hydrogen bond, *syn*-/*anti*- conformers, atoms numbering, and *d*_1_, *d*_2_ parameters assignment. 2-deoxyribose is represented by spheres. dG^oxo^ is represented by G^o^.

The spatial structure of the DNA double helix is formed by “randomly” repeated A::T and G:::C canonical Base Pairs (BP) connected iteratively by phosphor diester bonds, which together with a sugar moiety form a sugar-phosphate backbone. Each AT and GC pair are formed by complementary bases connected by Watson–Crick Hydrogen Bonds (HB)—two in AT and three in GC pairs. Additionally, they are stabilized by an external hydration layer. The AT or GC pair in ds-DNA is solvated by 34 and 44 water molecules respectively [24]. It should be pointed out that only the external shape of the double helix is surrounded by a solvation layer. The situation is different when a single nucleotide/nucleoside pair is taken into consideration, as it is completely warped by water molecules. In both cases, solvation influences parameters such as stability, HBs, stacking energy, charge migration parameters, electronic properties, etc. In a few special cases (triplex, tetraplex), the nucleobases can bond together by another type of HB, such as Hoogsteen or reverse-Hoogsteen [25]. These interactions require changes in the purine base orientation relative to the ribose moiety from *anti* to *syn* [26]. The base rotation around the glycosidic bond allows the non-canonical NP formation between dA and dG^oxo^ (Figure 2). The total energy of the two hydrogen bonds in dA::dG^oxo^ was found to be 11.67 kcal, which was higher by 7.58 kcal than that assigned for a dA:dG mismatch and smaller by 5.97 kcal than that for the canonical Watson–Crick dC:::dG (Table 1). Moreover, the guanosine flip from the *anti* to *syn* position in the dA:dG nucleoside pair leads to increases in the two structural parameters *d*_1_ and *d*_2_, in comparison to dC:::dG and dC:::dG^oxo^ systems, as a consequence of the N7 of the dG and N1 of the dA lone electron pair steric interaction (Figure 2). Therefore, the mutual dA and dG position permit only one hydrogen bond formation between N6 of the dA and O6 of the dG atoms. The situation is different after a one-electron dG oxidation event, which can lead to dG^oxo^ formation [27,28]. Due to the presence of additional oxygen at positions C8 and N7, atom protonation 8-oxo-7,8-dihydro-2′-deoxyguanosine can form with 2′-deoxyadenosine two hydrogen bonds (Figure 2). These interactions lead to the closer proximity of dA and dG^oxo^ in the non-canonical nucleoside pair, i.e., dA::dG^oxo^. Therefore, *d*_1_ and *d*_2_ were assigned at almost the same level as for native dC:::dG pairs, as well as λ_R_, λ_Y_, and λ_3_. The selected structural parameters of the above-mentioned nucleoside pairs and their description are presented in Table 1.

### 2.2. Electronic Properties of Isolated Nucleoside Pairs

**Figure 3:**
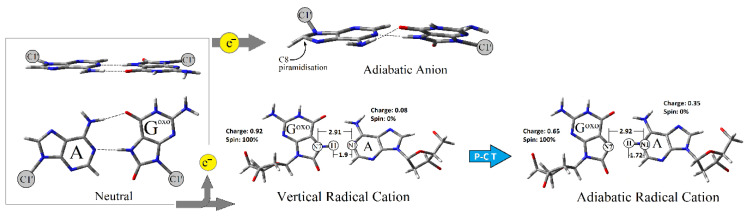
Graphical representation of proton charge transfer and C8 function pirymidization during the dA::dG^oxo^ ionization process.

The theoretical studies of Cauët at the MP2/6-31G(2d,p) level of theory have shown that the Vertical Ionization Potential (VIP) value of guanine (G) clusters decreases as the G number increases in the cluster formed by Gs [30]. Due to this, it can be expected that the G-rich part of the genome is a suitable area for dG^oxo^ formation [31]. In this study, the Adiabatic Ionization Potential (AIP) of dC:::dG^oxo^ was found to be lower than that calculated for dC:::dG by 0.14 eV (Table 2). The above results clearly indicate that the presence of dG^oxo^ in the system deepens the sink for the migrated radical cation (hole), thereby protecting the neighboring parts of the ds-DNA [32,33]. Surprisingly, the replacement of dC by dA in the nucleoside pair with dG^oxo^ leads to an adiabatic ionization potential decrease by an additional 0.37 eV in comparison with dC:::dG. Conversely, the mismatched NP dA:dG presented the highest adiabatic ionization potential (AIP) of all the investigated molecules, i.e., 6.03 eV. In this study, the following order of AIP was found: dA:dG>dC:::dG>dC:::dG^oxo^>dA::dG^oxo^ (Table 2). It is important to mention here that for dA::dG^oxo^, the proton transfer from N7 (dG^oxo^) to N1 (dA) was observed after adiabatic cation radical formation (Figure 3). (These results are in good agreement with those previously obtained by Sevilla for canonical BP [34].) The above is well-supported by the charge transfer notification between dA and dG^oxo^ of dA:dG^oxo^ (Table 2). In the vertical cation stage of the mentioned nucleoside pair, the positive charge accumulated mainly on the dG^oxo^ moiety (0.92), and after nuclear relaxation, a subsequent charge ratio of 65:35 was found for dA and dG^oxo^, respectively. Based on this observation, it can be predicted that the formed adiabatic radical cation can form a barrier (a “dam”) for the roving electron through the double helix. Conversely, for the other discussed molecules, the proton transfer was not observed. For the adiabatic radical cation form of the discussed molecules, a careful charge analysis elucidated the following distribution (in [a.u]): dA:dG—0.0/0.10 (dA/dG), dC::dG^oxo^—0.21/0.89 (dC/dG^oxo^), dC:::dG—0.20/0.80 (dC/dG). Subsequently, in each discussed case, almost 100% of spin density was found on the dG or dG^oxo^ moiety in both the vertical and adiabatic cation radical forms of the mentioned nucleoside pairs. The discussed data is laid out in Table 2.

**Figure 4:**
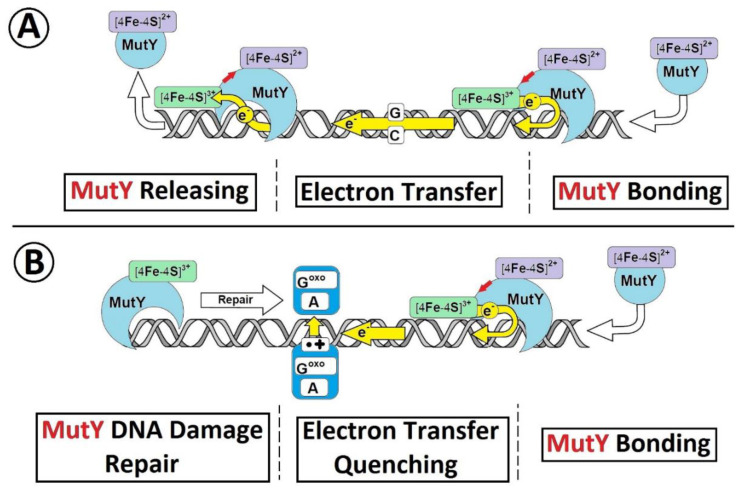
A schematic overview of [4Fe-4S] glycosylase (MutyH) DNA damage recognition under ds-DNA charge transfer mode. (**A**) The unimpaired electron transfer through double helix between two MutY proteins, (**B**) The electron transfer quenching between two MuitY when the dA::dG^oxo^ is present in ds-DNA structure [37].

The results of the theoretical studies presented in this article are in good agreement with the proposed mechanism of DNA damage recognition by MutY postulated and investigated experimentally by Barton and theoretically by Cox [17,35,36]. For this purpose, briefly MutY binds to the *ds*-DNA, and the [4Fe-4S]^2+^ cluster undergoes a one-electron oxidation to [4Fe-4S]^3+^. The ejected electron travels through the double helix until it is permanently trapped by dA::dG^oxo^, in its radical cation form. Due to the fact that the binding of MutY to ds-DNA in an oxidized form is 1000 times higher than in a reduced one, with the lack of electron (reverse process), enzyme cannot be dissociated from the double helix and, therefore, move to the place in which the electron was settled Figure 4 [18,37].

**Figure 5:**
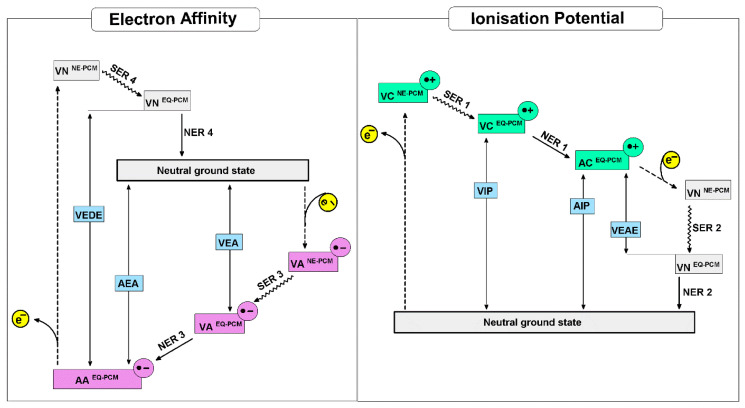
Graphical representation of nucleoside pairs electronic parameters and energetic states discussed in the manuscript [41].

In light of the above, the electronic parameters of a glycosylase substrate, i.e., dA:dG and dA::dG^oxo^, ionization potential and electron affinity are the crucial parameters for the recognition process explanation/description. In the context of the electronic properties, the loss of an electron by a molecule can be described as: (1) an electron ejected without a molecule and solvent layer rearrangement, i.e., non-Equilibrium state, (VIP^NE-**PCM**^); (2) relaxation of the solvent layer without molecular geometry changes, i.e., equilibrium state, (VIP^EQ-**PCM**^); (3) ground state achievement by solvent and molecule, denoted as the adiabatic ionization potential. In this study, the Polarizable Continuum Model (PCM) was used for the solvent environment description [38]. As for radical cation stability, the capability of electron uptake by the radical cation can be described by the Vertical Electron Attachment Energy (VEAE) in non-equilibrium (VEAE^NE-**PCM**^) and equilibrium (VEAE^EQ-**PCM**^) modes. Moreover, the energy discrepancy between the equilibrium vertical state and adiabatic corresponds to the “work movement”, which must be performed by atoms in a molecule, denoted as Nuclear Relaxation Energy (NER). The relaxation energy of the solvation layer can be described as the difference between energies obtained for molecule in a vertical state using non-equilibrium and equilibrium PCM modes (SRE: Solvent Relaxation Energy). A graphical representation of the above process has been shown in Figure 5.

The lowest VIP of all the investigated molecules was assigned to dA::dG^oxo^ in both the non-equilibrium state and equilibrium state modes. However, for dC:::dG, dA::dG^oxo^, and dC:::dG^oxo^ molecules, the VIP^NE-PCM^ and VIP^EQ-PCM^ values were found at the same level (around 6.0 eV). However, for dA:dG, the VIP^NE-PCM^ value was found to be 0.01 eV higher than that of VIP^EQ-PCM^ (Table 2). The above indicates that in the mismatched nucleoside pair case, the solvent layer relaxation process is preferred before the molecule achieves an adiabatic cation form, contrary to the other discussed NPs. The adiabatic radical cation state formation process requires a spatial molecule geometry rearrangement, after the electron loss. The nuclear relaxation energy, denoted as NER 1, was calculated as the difference between the VIP^EQ-PCM^ and AIP of the discussed molecules presented the following order: dA::dG^oxo^ > dA:dG > dC:::dG^oxo^ > dC:::dG (Table 2). Therefore, since the formation of a dA::dG^oxo^ radical cation is the most privileged process, the NER 1 obtained for this nucleoside pair was at least 1.5 times higher than that denoted for the other discussed systems.

Due to the nature of charge migration through the double helix, which is an iterative process of electron loss and electron uptake by the nucleobases, the radical cation’s propensity for electron attachment is the important parameter of this process and therefore for DNA lesion recognition by MutY. As previously, the additional electron appearing in the molecule structure forces changes in the solvent layer due to the vertical neutral state formation, which after the relaxation yields a ground neutral state. Therefore, the difference between the VEAE^NE-PCM^ and VEAE^EQ-PCM^ values, i.e., SER 2, should be discussed. The following order of SER 2 was found: dA:dG > dC:::dG^oxo^ ~ dC:::dG > dA::dG^oxo^ (Table 2). These results are in good agreement with the NER 2 value (please see Figure 5), which indicated that the formation of the neutral ground state from a vertical one is privileged for dA::dG^oxo^ (NER-2 = 0.71 eV) and less preferred by dA:dG (NER 2 = 0.31 eV) (Table 2).

Due to the relatively short life of the dA::dG^oxo^ radical cation, it is interesting to look at the question of why an electron roving through ds-DNA can be effectively captured by this type of damage in its neutral ground state. Therefore, if MutY glycosylase scanning ds-DNA via the electron transfer mechanism, the Electron Affinity (EA) of targeted dA::dG^oxo^ and dA:dG should be taken into consideration. Electron affinity has been defined as the ability of a molecule to adopt an extra electron. Therefore, as it shown on Figure 4, the EA can be discussed in three modes: Vertical Electron Affinity in a Non-Equilibrium solvent state (VEA^NE-PCM^), Vertical Electron Affinity in an Equilibrium solvent state (VEA^EQ-PCM^), and as Adiabatic Electron Affinity (AEA). For the investigated molecules, the following order of VEA^EQ-PCM^ and AEA was noted: dA:dG < dA::dG^oxo^ < dC:::dG < dC:::dG^oxo^ (Table 2). Moreover, for dA:dG (the mismatched nucleoside pair), the highest difference between VEA^NE-PCM^ and VEA^EQ-PCM^ was found, i.e., 0.01 eV; for the others, these value were negligible. This could suggest that in the initial state of dA:dG, an electron attachment requires a solvation layer rearrangement. Subsequently, the nuclear reorganization energy NER 3 of dA:dG, which is the indicator of geometry reorganization, was found to be the lowest, i.e., 0.29 eV. For dC:::dG^oxo^, dC:::dG^oxo^, and dC:::dG, the obtained values were almost 1.5 times higher than for dA:dG, i.e., ~0.48 eV (Table 2). The stability of the formed radical anion during the charge transfer process can be viewed as the ability of the electron to “escape” from a negatively ionized molecule. This can be denoted by Vertical Electron Detachment Energy (VEDE), which describes the energy necessary for extra electron removal from an anion. The higher the VEDE, the more stable the anion. The lowest value of VEDE^NE-PCM/EQ-PCM^ was denoted for the dA:dG system (1.60 eV), while the others were found at a level of around 2.58 eV. Moreover, in all cases, no notable differences between VEDE^NE-**PCM**^ and VEDE^EQ-**PCM**^ were assigned (Table 2). Based on the above it can be predicted that mismatched NP formed a less stable anion, therefore an electron can easily leave the discussed system and move forward through the double helix until it reduces the [4Fe-4S]^3+^ cluster and realizes the MutY from the double helix. Additionally, this was supported by the lowest dA:dG NER_4_ value assignment among all the investigated molecules, i.e., 0.32 eV. The NER_4_ for dC:::dG and dC:::dG^oxo^ was found at the level of 0.58 eV. The NER_4_ parameter described the energetic requirements for a nucleus rearrangement after electron loss by the anion. In contrast to dA:dG, the NER_4_ of dA::dG^oxo^ was the highest among all the discussed molecules, i.e., 0.98 eV, and indicates the significant geometrical difference between the anion and neutral states. Moreover, the following vertical anion dipole moments of dA:dG and dA::dG^oxo^ were noted: 26[D] and 12[D] respectively, as opposed to their neutral forms 9[D] and 12[D]. These observations follow Adamowicz’s work and indicate that the dA:dG anion shows a dipole-bond character instead of dA:::dG^oxo^, which forms the valence type [39,40]. However, both molecules after adiabatic anion formation adopted a dipole moment at the same level, i.e., 25 and 26 [D] for dA:dG and dA::G^oxo^, respectively. Based on the above, it can be concluded that the dA::dG^oxo^ nucleoside pair can form a stable adiabatic anion. A careful spin distribution analysis of the discussed nucleoside pairs indicates that in each investigated case, spin density is mainly accumulated on the adenine or cytosine moiety (Table 2) in vertical and adiabatic states. However, after the nuclear relaxation process (NER_3_) the spin density increases (by 16%) exclusively on the adenine C8 atom of the dA::dG^oxo^ pair, which leads to mentioned carbon atom piramidisation (*sp*^3^ hybridisation) (Figure 3). This indicates that C8 will be most prone to a reaction with other radicals like hydroxyl (^●^OH). A parallel negative charge distribution analysis shows that in the case of dC:::dG and dC:::G^oxo^, a negative charge was dispersed over both bases of the nucleoside pair, while in the cases of dA:dG and dA:::dG^oxo^ it was found mainly on the adenine moiety in the vertical and adiabatic states (Table 2).

VC^NE-PCM^[•+]—Vertical Cation in Non-Equilibrium PCM mode, VC^EQ-PCM^[•+]—in EQuilibrium PCM mode; AC^EQ-PCM^[•+]—Adiabatic Cation in equilibrium mode; VNC^NE-PCM^—Vertical Neutral formed from Cation in non-equilibrium PCM mode and VNC^EQ-PCM^—in equilibrium PCM mode; VA^NE-PCM^[•−]—Vertical Anion in non-equilibrium PCM mode, VA^EQ-PCM^[•−]—in equilibrium PCM mode; AA^EQ-PCM^[•−]—Adiabatic Cation in equilibrium PCM mode VNA^NE-PCM^—Vertical Neutral formed from Anion in non-equilibrium PCM and VNA^EQ-PCM^—in equilibrium PCM mode.

VIP—Vertical Ionisation Potential, AIP—Adiabatic Ionisation Potential, VEAE—Vertical Electron Attachment Energy, NER 1—the Nuclear Relaxation Energy 1 difference between the energies of vertical and adiabatic cation, NER-2—the Nuclear Relaxation Energy 2 difference between the energies of vertical neutral state formed from cation and adiabatic state of the molecule, SER 1—Solvent Relaxation Energy 1 the energies of difference between vertical cation in non-equilibrium PCM and in equilibrium PCM mode, SER 2—Solvent Relaxation Energy 2 the difference between the energies of vertical neutral states formed from cation in non-equilibrium and in equilibrium PCM mode. VEA—Vertical Electron Affinity, AIP—Adiabatic Electron Affinity, VEDE—Vertical Electron Detachment Energy, NER 3,—the Nuclear Relaxation Energy 3 difference between the energies of vertical and adiabatic anion, NER 4—the Nuclear Relaxation Energy 4 difference between the energies of vertical neutral state formed from the anion and adiabatic state of the molecule, SER 3—Solvent Relaxation Energy 3 the difference between the energies of vertical anion in non-equilibrium and in equilibrium PCM mode, SER 4—Solvent Relaxation Energy 4—the difference between the energies of vertical neutral stats formed from anion in non-equilibrium and in equilibrium PCM mode.

### 2.3. Molecular Orbital Analysis of dA::dG^oxo^ and dA:dG Pairs

According to Koopmans’ theorem, the negative energy of high occupied orbital (HOMO) and low unoccupied molecular orbital (LUMO) should be related to the vertical ionization energy and vertical electron affinity energy, respectively [42]. After a single electron loss, the HOMO of a neutral molecule was split into β-LUMO and α-SOMO. Therefore, during one electron oxidation process, a single occupied molecular orbital should be the HOMO according to the aufbau principle. However, the recent studies of Sevilla et al. have shown that in the case of nucleic bases, the odd electron orbital lies below the HOMO [43].

In these studies, the effect of solvent relaxation was negligible for all the investigated orbitals (Table S2). The average value of SER-1 was found to be below 0.072 eV for α-MO and 0.012 eV for β-MO in both the vertical cation and vertical anion radical states. In this work, the HOMO of dA::dG^oxo^ and dC:::dG^oxo^ showed lower energy than dC:::dG and dA:dG in their neutral forms (see Table S2 and Figure S1). The orbital analysis elucidates that in the vertical radical cation state α-SOMO is buried under the HOMO in each investigated nucleoside pair. However, for dC::dG^oxo^, dC:::dG and dA::dG^oxo^, it was localized just under the HOMO, while for dA:dG α-SOMO, it was assigned as the third orbital below HOMO. After vertical radical cation spatial geometry relaxation and adiabatic radical cation formation, orbital rearmament was observed. α-SOMO of dA::dG^oxo^ and dC:::dG^oxo^ becomes HOMO, while in the remaining case, the molecules were still buried under HOMO, but just below. These observations clearly indicate that nucleoside pairs composed by dG^oxo^ are predisposed to one-electron oxidation and stable radical cation formation. Moreover, in the case of dA::dG^oxo^, the rearrangement of the mentioned orbitals was accompanied by a proton-electron transfer process. This phenomenon was previously noted and confirmed by the careful studies of Sevilla, who observed the SOMO-HOMO discrepancy/inversion as characteristic of a molecule but not as a calculation method property [43]. This situation was similar when the extra electron adoption by a molecule was taken into consideration. The following LUMO energy order of a neutral state was found: dC::dG^oxo^ > dC:::dG >dA::dG^oxo^ > dA:dG. The same energy order was found for α-SOMO of vertical and adiabatic anion radical states of the discussed nucleoside pairs. However, the negative α-SOMO energy increased after the vertical→adiabatic state conversion (Table S2). It is important to mention that in all the cases, α-SOMO lies just over HOMO. Due to the fact that the α-SOMO energy was found to be lower for dA::dG^oxo^ than for the dA:dG vertical and adiabatic radical anion, it could be postulated that dA::dG^oxo^ can be the privileged point for extra electron capturing. Therefore, this lesion recognition by MutY should be faster than that observed for the mismatched dA:dG pair when the electron transfer DNA damage searching mechanism is taken into consideration.

## 3. Materials and Methods

All calculations of solely base pairs designated as dC:::dG, dC:::dG^oxo^, dA:dG, dA::dG^oxo^ were performed with density functional theory (DFT) using the M062x functional [44]. For all geometry calculations/optimizations, the D95+* basis set was used [45]. For the characterization of stationary point energies and the electronic properties of all the investigated nucleoside pairs, harmonic vibrations and energies were calculated at the M062x/6-31++G** level. For each structure being a minimum, no imaginary frequency was found.

All calculations were performed in the condensed phase using Tomasi’s polarizable continuum model (PCM) [38]. For all the optimized structures, a charge and spin analysis was achieved using Hirshfeld methodology at the M062x/6-31++G(d,p) level of theory [46]. The value of spin and charge was calculated for heavy atoms summed to one, and given in a.u. The electronic properties of molecules were calculated as described previously: for further details, please see [47,48]. The solvent effect was taken considered in two modes, following previously described methodology: non-equilibrium PCM (NE-PCM) and equilibrium PCM (EQ-PCM) [49].

All the above calculations were performed with the Gaussian software package [50].

## 4. Conclusions

In conclusion, the dA::dG^oxo^ pair appearing in the nucleic ds-DNA may lead to a mutation in genetic information if not repaired. Depending on the dG^oxo^ source, a AT→GC and GC→AC transversion can be observed. As a result, the two main glycosylases developed during the evolution of OGG1 and MutY. While the first one effectively removes G^oxo^, the second protein recognizes adenine as part of the dA::dG^oxo^ and dA:dG pairs. In line with S.S. David’s proposition, MutY can recognize DNA damage site by an electron transfer between two red-ox proteins.

In this article, the structural and electronic properties of the model nucleoside pairs dA:dG, dC:::dG^oxo^, dC:::dG, dA::dG^oxo^ were taken into theoretical consideration in the aqueous phase. The influence of solvent layer relaxation on the above was also discussed.

It was found that the isolated dA::dG^oxo^ shows the lowest vertical and adiabatic ionization potential energies for all the discussed molecules. For the above nucleoside pair, the proton charge transfer was exclusively observed after one-electron oxidizing.

The one-electron reduction of nucleoside pairs elucidated a higher vertical and adiabatic electron affinity for dA::dG^oxo^ than for dA:dG. Moreover, for isolated dA::dG^oxo^, pyramidization of the C8 atom of dG^oxo^ was observed. This indicates that C8 is most prone to a reaction with other radicals like hydroxyl (^●^OH).

The molecular orbital analysis shows that α-SOMO energy was lower for dA::dG^oxo^ than for that of the dA:dG vertical/adiabatic radical cation and anion. It can thus be postulated that dA::dG^oxo^ is the privileged point for extra electron capturing. The above nucleoside pair recognition by MutY should be faster than that observed for a mismatched dA:dG pair when the “electron transfer DNA damage searching” mechanism is taken into consideration.

The obtained results are in good agreement with previous experimental data described by S.S. David, which have shown that dA::dG^oxo^ is recognized and removed ~6–10 times faster than dA:dG.

## Figures and Tables

**Table 1:** Selected structural parameters of nucleoside pairs and hydrogen bond (HB) energy in kacl/mol. Distance ***d*_1_**: C^1′^_(dA/dC)_–C^1′^_(dG/dG_^oxo^_)_, ***d*_2_**: N^9/1^_(dA/dC)_–N^9^_(dG/dG_^oxo^_)_, angles **λ_R_**: N^9/1^_(dA/dC)_–C^1′^_(dA/dC)_–C^1′^_(dG/dG_^oxo^_)_, **λ_Y_**: N^9^_(dG/dG_^oxo^_)_–C^1′^_(dG/dG_^oxo^_)_–C^1′^_(dA/dC)_, dihedral angle **λ_3_**: N^9/1^_(dA/dC)_–C^1′^_(dA/dC)_–C^1′^_(dG/dG_^oxo^_)_–N^9^_(dG/dG_^oxo^_)_ [29].

System	HB Length [Å]			HB Energy	*d*_1_[Å]	*d*_2_[Å]	λ_R_[°]	λ_Y_[°]	λ_3_[°]
	HB1	HB2	HB3						
dC:::dG	2.89	2.94	2.84	−17.55	10.77	9.00	52.98	51.94	−6.01
dC:::dG^oxo^	2.87	2.91	2.83	−18.27	10.71	8.99	52.41	54.48	−6.69
dA:dG	2.91	4.03		−4.09	12.57	10.27	43.10	30.76	−8.77
dA::dG^oxo^	2.91	2.83		−11.67	10.76	8.87	53.89	43.97	−5.35
Reference Parameters of ds-DNA [29]									
T::dA	3.05	2.96			10.7		54.5	54.5	
dC:::dG	2.87	3.00	3.00		10.8		54.2	54.5	

**Table 2:** Electronic properties in eV of the discussed nucleoside pairs as well as charge and spin distribution calculated at the M062x/6-31++G** level of theory in the aqueous phase. (The abbreviations of the parameters are given in the Figure 5 caption.).

System	Electronic Properties in [eV]							
	VIP^NE-PCM^	VIP^EQ-PCM^	AIP	VEA^NE-PCM^	VEA^EQ-PCM^	AEA*	VEAE^NE/EQ-PCM^	VEDE^NE/EQ-PCM^
dC:::dG	6.12	6.12	5.77	1.51	1.51	2.00	2.58/2.57	5.44/5.43
dC:::dG^oxo^	6.01	6.01	5.63	1.53	1.53	2.02	2.61/2.61	5.26/5.26
dA:dG	6.45	6.44	6.03	0.99	1.00	1.28	1.61/1.60	5.71/5.71
dA::dG^oxo^	6.00	6.00	5.26	1.10	1.10	1.57	2.55/2.55	4.55/4.55
	**SER 1**	**NER 1**	**SER 2**	**NER 2**	**SER 3**	**NER 3**	**SER 4**	**NER 4**
dC:::dG	0.00	0.35	0.00	0.34	0.00	0.49	0.00	0.58
dC:::dG^oxo^	0.00	0.37	0.00	0.37	0.00	0.49	0.00	0.59
dA:dG	0.01	0.42	0.01	0.31	0.01	0.29	0.00	0.32
dA::dG^oxo^	0.00	0.73	0.00	0.71	0.00	0.47	0.00	0.98
**Nucleoside Pair**								
	**dC:::dG**		**dC:::dG^oxo^**		**dA:dG**		**dA::dG^oxo^**	
	**dC**	**dG**	**dC**	**dG^oxo^**	**dA**	**dG**	**dA**	**dG^oxo^**
**Form**	**Charge distribution** [a.u]							
Neutral	0.16	−0.16	0.18	−0.18	−0.11	0.11	0.04	−0.04
VC^EQ-PCM^	0.20	0.80	0.21	0.79	−0.09	1.09	0.08	0.92
AC	0.29	0.71	0.29	0.71	0.07	0.93	0.65	0.35
VA^EQ-PCM^	−0.77	−0.23	−0.76	−0.24	−1.09	0.09	−0.91	−0.09
AA	−0.68	−0.32	−0.66	−0.34	−1.07	0.07	−0.90	−0.10
VNC^EQ-PCM^	0.25	−0.25	0.25	−0.25	−0.09	0.09	0.59	−0.59
VNA^EQ-PCM^	0.26	−0.26	0.28	−0.28	−0.08	0.08	0.08	−0.08
Form	Spin Distribution [a.u]							
VC^EQ-PCM^	0.00	1.00	0.20	0.80	0.00	1.00	0.00	1.00
AC	0.00	1.00	0.29	0.71	0.00	1.00	0.00	1.00
VA^EQ-PCM^	0.98	0.02	0.99	0.01	0.99	0.01	0.99	0.01
AA	0.99	0.01	0.99	0.01	1.00	0.00	1.00	0.00

## Supplementary Materials

The following are available online, Table S1: The energies (in Hartree) as well as dipole moment (in Debye) of Neutral, Vertical Cation, Adiabatic Cation and Vertical Neutral forms of nucleoside pairs calculated at the M062x/6-31+G** level of theory in the aqueous phase. NE-PCM: non-equilibrium polarizable continuum model, EQ-PCM: equilibrium polarizable continuum model. Table S2: Configuration of α- and β- molecular orbitals (α-MO, β-MO) all energies are given in eV of dA::dG^oxo^, dA:dG, dC:::dG^oxo^, dC:::dG calculated at the M062x/6-31++G** level of theory in the aqueous phase. NE: non- and EQ: equilibrium PCM mode, H: HOMO, L: LUMO, O: other orbitals, S: SOMO. Figure S1: Calculated molecular orbital as well as spin density of optimized geometry of adiabatic: neutral, cation, anion and vertical: cation, anion and neutral of dA::G^oxo^, dA:dG, dC:::dG, dC:::dG^oxo^ base pairs. All molecular energies are given in eV calculated at the M062x/6-31+G** level of theory in the aqueous phase. Abbreviations: H—Highest Occupied Molecular Orbital, L—Low Occupied Molecular Orbital, S—Single Occupied Molecular Orbital, NE—non-equilibrated polarizable continuum model, EQ—equilibrated polarizable continuum model.
