# Supplementary material for: The Electronic Property Differences between dA::dG and dA::dGoxo. A Theoretical Approach
Source: Molecules. 2020 Aug 23;25(17):3828. doi: 10.3390/molecules25173828 (PMC7503971; doi:10.3390/molecules25173828)
Supplement: Supplementary file 1 [file molecules-25-03828-s001.zip › Fig S1.pdf]

**Figure S1.**

Calculated molecular orbital as well as spin density of optimized geometry of adiabatic: neutral, cation, anion and vertical: cation, anion and neutral of dA::G$^{oxo}$, dA:dG, dC:::dG, dC:::dG$^{oxo}$ base pairs. All molecular energies have been given in eV calculated at M062x/6-31+G** level of theory in aqueous phase. Abbreviation: H - Highest Occupied Molecular Orbital, L - Low Occupied Molecular Orbital, S - Single Occupied Molecular Orbital, NE – non-equilibrated polarizable continuum model, EQ – equilibrated polarizable continuum model.

| dA::dG$^{oxo}$ | Vertical Cation (EQ/NE) [•+] | | Cation [•+] | | Vertical Anion (EQ/NE) [•-] | | Anion [•-] | |
|---|---|---|---|---|---|---|---|---|
| MO | α-MO | β-MO | α-MO | β-MO | α-MO | β-MO | α-MO | β-MO |
| -0.15 ($O^{142}$) 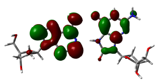 | | | | | | -0,18/-0.18 ($L^{141}$) 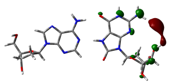 | | -0.17 ($L^{141}$) 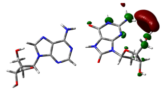 |
| -0.20 ($L^{141}$) 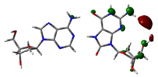 | | -4.92/-4.91 ($L^{140}$) 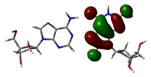 | | -3.45 ($L^{140}$) 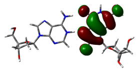 | -2,12/-2.12 ($S^{141}$) 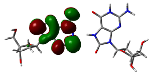 | | -3.66 ($S^{141}$) 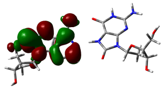 | |
| -7.07 ($H^{140}$) 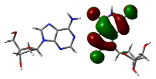 | -7.79/-7.79($H^{140}$) 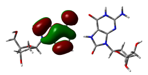 | -7.79/-7.79 ($H^{139}$) 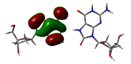 | -7.37($S^{140}$) 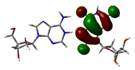 | | -6,90/-6.91 ($H^{140}$) 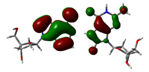 | -6,35/-6.35($H^{140}$) 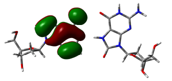 | -6.98 ($H^{140}$) 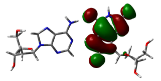 | -6.69 ($H^{140}$) 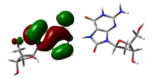 |
| -7.67 ($O^{139}$) 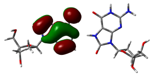 | -8,92/-8.91($S^{139}$) 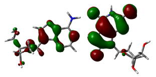 | | -8,38($H^{139}$) 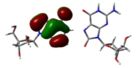 | -8,38 ($H^{139}$) 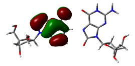 | -6.98/-6.98 ($O^{139}$) 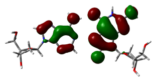 | -6.96/-6.96 ($O^{139}$) 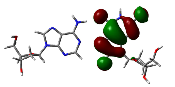 | -7.37 ($O^{139}$) 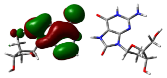 | -6.98 ($O^{139}$) 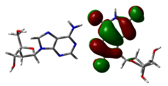 |
| **Spin** | 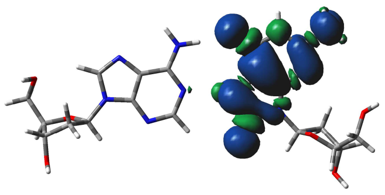 | | 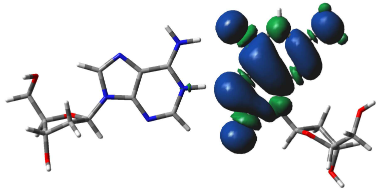 | | 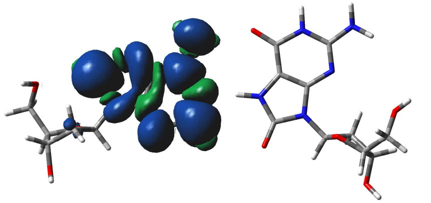 | | 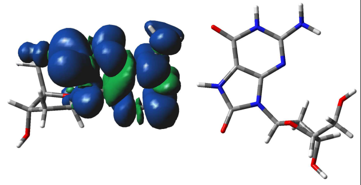 | |

| **dA:dG** | **Vertical Cation [•+]** | | **Cation [•+]** | | **Vertical Anion [•-]** | | Anion [•-] | |
|---|---|---|---|---|---|---|---|---|
| **MO** | **α-MO** | **β-MO** | **α-MO** | **β-MO** | **α-MO** | **β-MO** | **α-MO** | **β-MO** |
| -0.08 ($O^{138}$) 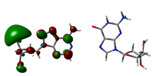 | | | | | | -0,16/-0.15 ($L^{137}$) 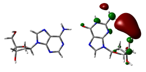 | | -0,15 ($L^{137}$) 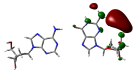 |
| -0.18 ($L^{137}$) 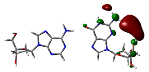 | | -5,36/-5.37 ($L^{136}$) 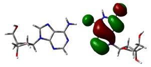 | | -4,63 ($L^{136}$) 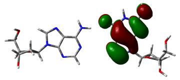 | -2,03/-2.03 ($S^{137}$) 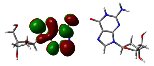 | | -2,64 ($S^{137}$) 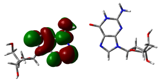 | |
| -7.49 ($H^{136}$) 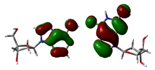 | -7.60/-7.60($H^{136}$) 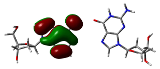 | -7.60/-7.60($H^{135}$) 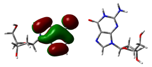 | -7.62 ($H^{136}$) 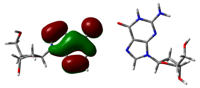 | -7.62($H^{135}$) 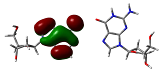 | -6,80/-6.08 ($H^{136}$) 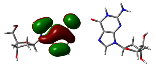 | -6,25/-6.25 ($H^{136}$) 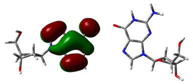 | -7,44 ($H^{136}$) 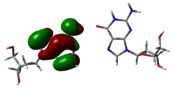 | -7,44 ($H^{136}$) 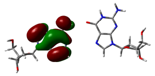 |
| -7.56 ($O^{135}$) 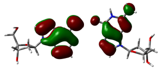 | -8,82/-8.82($O^{135}$) 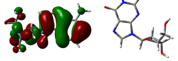 | -8,82/-8.82($O^{134}$) 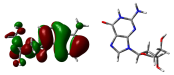 | -8,59 ($S^{135}$) 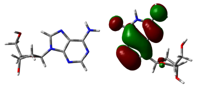 | | -7.46/-7.46 ($O^{135}$) 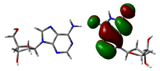 | -7.46/-7.46 ($O^{135}$) 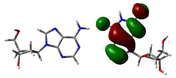 | -8.18 ($O^{135}$) 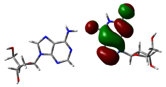 | -7.89 ($O^{135}$) 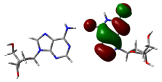 |
| | -9,07/-9.08($O^{134}$) 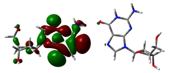 | -9,07/-9.07($O^{133}$) 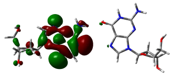 | | | | | | |
| | -9,33 ($S^{133}$) 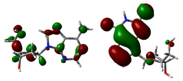 | | | | | | | |

| | | | | | | | | |
|---|---|---|---|---|---|---|---|---|
| Spin | 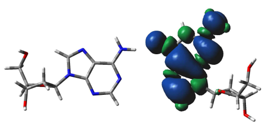 | | 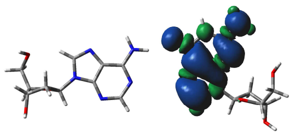 | | 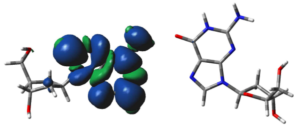 | | 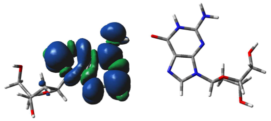 | |
| **dC:::dG$^{oxo}$** | **Vertical Cation [•+]** | | **Cation [•+]** | | **Vertical Anion [•-]** | | **Anion [•-]** | |
| **MO** | **α-MO** | **β-MO** | **α-MO** | **β-MO** | **α-MO** | **β-MO** | **α-MO** | **β-MO** |
| -0.17 ($O^{136}$ ) 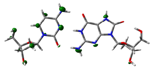 | | | | | | -0.14/-0.14 ($L^{135}$) 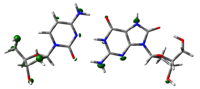 | | -0,13 ($L^{135}$) 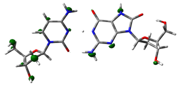 |
| -0.45 ($L^{135}$) 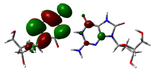 | | -4.92/-4.93 ($L^{134}$) 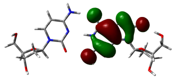 | | -4.15 ($L^{134}$) 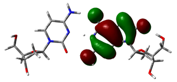 | -2.66/-2.66($S^{135}$) 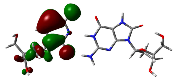 | | -3.75($H^{135}$) 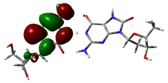 | |
| -7.07 ($H^{134}$) 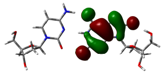 | -8,29/-8.29($H^{134}$) 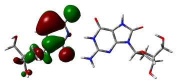 | -8,29/-8.29 ($H^{133}$) 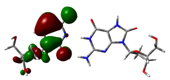 | -8,23($S^{134}$) 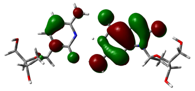 | | -6,96/-6.96($H^{134}$) 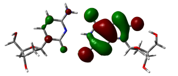 | -6.75/-6.75 ($H^{134}$) 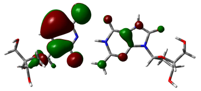 | -6,94($S^{134}$) 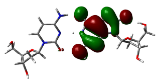 | -6.94 ($H^{134}$) 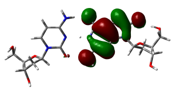 |
| -8,20 ($O^{133}$) 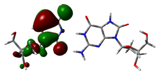 | -8,95/-8.92($S^{133}$) 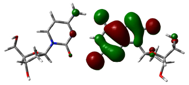 | | -8,40($H^{133}$) 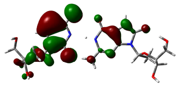 | -8,38 ($H^{133}$) 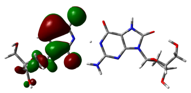 | -7.30/-7.30 ($O^{133}$) 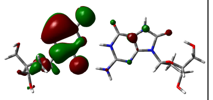 | -6.98/-6.98 ($O^{133}$) 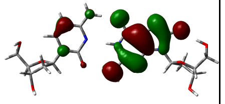 | | |

| **Spin** | 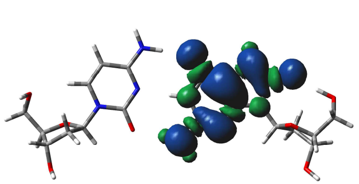 | | 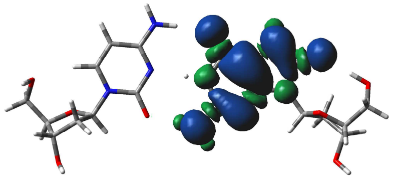 | | 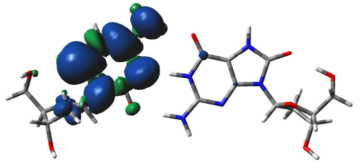 | | 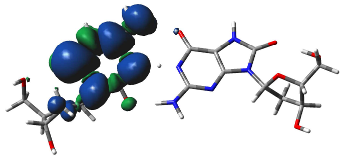 | |
|---|---|---|---|---|---|---|---|---|
| **dC:::dG** | **Vertical Cation [•+]** | | **Cation [•+]** | | **Vertical Anion [•-]** | | **Anion [•-]** | |
| **MO** | **α-MO** | **β-MO** | **α-MO** | **β-MO** | **α-MO** | **β-MO** | **α-MO** | **β-MO** |
| -0.15(O$^{132}$ ) | | | | | | -0,12/-0.12 (L$^{131}$) | | -0,11 (L$^{131}$) |
| 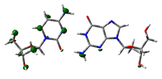 | | | | | | 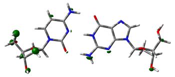 | | 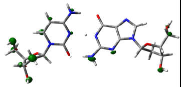 |
| -0.42 (L$^{131}$) | | -5.05/-5.05 (L$^{130}$) | | -4,21 (L$^{130}$) | -2,64/-2.64 (S$^{131}$) | | -3,72(H$^{131}$) | |
| 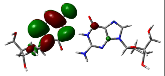 | | 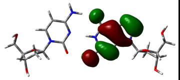 | | 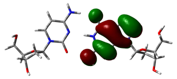 | 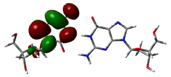 | | 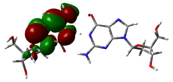 | |
| -7.18 (H$^{130}$) | -8,25/-8.28(H$^{130}$) | -8,27/-8.28 (H$^{129}$) | -8,27(H$^{130}$) | | -7,06/7.06 (H$^{130}$) | -6,73/-6.73 (H$^{130}$) | -7,06(S$^{130}$) | -7,06 (H$^{130}$) |
| 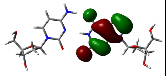 | 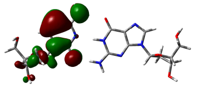 | 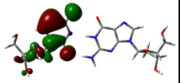 | 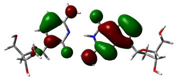 | | 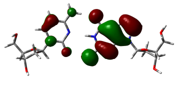 | 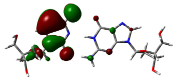 | 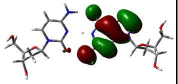 | 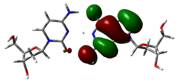 |
| -8,18 (O$^{129}$) | -9,03/-9.03(S$^{129}$) | | -8.40(S$^{129}$) | -8,75 (H$^{129}$) | -7.28/-7.28 (H$^{129}$) | --7.09/7.09 (H$^{129}$) | -7,69(S$^{129}$) | -7,02(H$^{129}$) |

| | | | | | | | | |
|---|---|---|---|---|---|---|---|---|
| 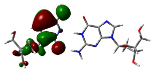 | 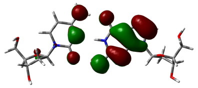 | | 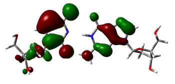 | 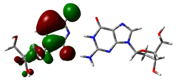 | 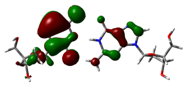 | 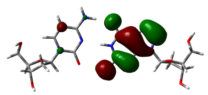 | 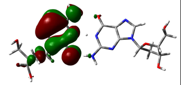 | 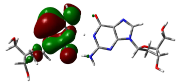 |
| | -9.04/-9.04($O^{128}$) 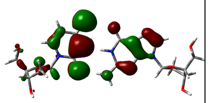 | -9,03/-9.03 ($O^{128}$) 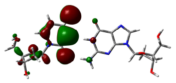 | -9,18($O^{128}$) 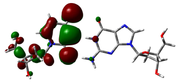 | -9,18 ($O^{128}$) 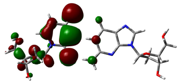 | | | | |
| Spin | 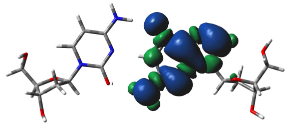 | | 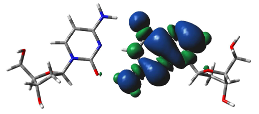 | | 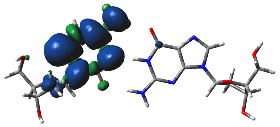 | | 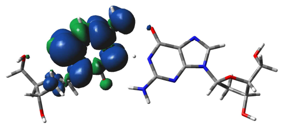 | |
