# Supplementary material for: The Electronic Property Differences between dA::dG and dA::dGoxo. A Theoretical Approach
Source: Molecules. 2020 Aug 23;25(17):3828. doi: 10.3390/molecules25173828 (PMC7503971; doi:10.3390/molecules25173828)
Supplement: Supplementary file 1 [file molecules-25-03828-s001.zip › Table S1.pdf]

Table S1

The energies (in Hartree) as well as dipole moment (in Debys) of Neural, Vertical Cation, Adiabatic Cation and Vertical Neutral forms of nucleoside pairs and *ds*-tetramers calculated at the M062x/6-31+G** level of theory in the aqueous phase. NE-PCM: non-equilibrated polarizable continuum model, EQ-PCM: equilibrated polarizable continuum model.

Nucleoside Pairs						
System	Energy	Dipol Moment	Energy	Dipol Moment	Energy	Dipol Moment
	Neutral		Vertical Cation NE-PCM		Vertical Cation EQ-PCM	
dG:::dC	-1778,994151	7,86	-1778,769269	7,58	-1778,769184	7,58
dC:::dGoxo	-1854,227234	17,61	-1854,006449	6,80	-1854,006411	6,80
dA:dG	-1851,332567	12,01	-1851,095484	28,77	-1851,095721	28,77
dA::dGoxo	-1926,569674	9,13	-1926,349366	22,18	-1926,349360	22,18
	Adianbatic Cation		Vertical Neutral from Cation NE-PCM		Vertical Neutral from Cation EQ-PCM	
dG:::dC	-1778,781997	6,32	-1778,981731	9,76	-1778,981708	9,76
dC:::dGoxo	-1854,020192	7,75	-1854,213589	18,68	-1854,213567	18,68
dA:dG	-1851,11099	29,63	-1851,3207910	11,45	-1851,3210036	11,45
dA::dGoxo	-1926,376311	14,42	-1926,543595	9,42	-1926,5436002	9,42
	Vertical Anion NE-PCM		Vertical Anion EQ-PCM			
dG:::dC	-1779,049641	16,36	-1779,049536	16,36		
dC:::dGoxo	-1854,283494	10,05	-1854,283494	10,05		
dA:dG	-1851,369047	26,54	-1851,369285	26,54		
dA::dGoxo	-1926,610044	19,252	-1926,610018	19,25		
	Adianbatic Anion		Vertical Neutral from Anion NE-PCM		Vertical Neutral from Anion EQ-PCM	
dG:::dC	-1779,067558	14,28	-1778,972907	10,37	-1778,972951	10,37
dC:::dGoxo	-1854,301521	9,43	-1854,205422	20,36	-1854,205503	20,36
dA:dG	-1851,379672	24,66	-1851,320633	8,11	-1851,320754	8,10
dA::dGoxo	-1926,627357	26,19	-1926,533484	8,89	-1926,533555	8,89
