# Supplementary material for: The Electronic Property Differences between dA::dG and dA::dGoxo. A Theoretical Approach
Source: Molecules. 2020 Aug 23;25(17):3828. doi: 10.3390/molecules25173828 (PMC7503971; doi:10.3390/molecules25173828)
Supplement: Supplementary file 1 [file molecules-25-03828-s001.zip › Table S2.pdf]

Table 2SM. Configuration of α- and β- molecular orbitals (α-MO, β-MO); all energies are given in eV of dA::dG^{oxo}, dA:dG, dC:::dG^{oxo}, dC:::dG calculated at the M062x/6-31++G** level of theory in the aqueous phase. NE: non- and EQ: equilibrium PCM mode, H: HOMO, L: LUMO, O: other orbitals, S: SOMO.

**Neutral**	**Vertical Cation (EQ/NE) [•+]**		**Cation [•+]**		**Vertical Anion (EQ/NE) [•-]**		**Anion [•-]**	
**MO**	**α-MO**	**β-MO**	**α-MO**	**β-MO**	**α-MO**	**β-MO**	**α-MO**	**β-MO**
				**dA::dG^{oxo}**				
-0.15(O^{142})						-0.18/-0.18(L^{141})		-0.17(L^{141})
-0.20 (L^{141})		-4.92/-4.91(L^{140})		-3.45(L^{140})	-2.12/-2.12(S^{141})		-3.66(S^{141})	
-7.07 (H^{140})	-7.79/-7.79(H^{140})	-7.79/-7.79 (H^{139})	-7.37(S^{140})		-6.90/-6.91(H^{140})	-6.35/-6.35(H^{140})	-6.98(H^{140})	-6.69(H^{140})
-7.67 (O^{139})	-8.92/-8.91(S^{139})		-8.38(H^{139})	-8.38(H^{139})	-6.98/-6.98(O^{139})	-6.96/-6.96(O^{139})	-7.37(O^{139})	-6.98(O^{139})
				**dA:dG**				
-0.08 (O^{138})						-0.16/-0.15(L^{137})		-0.15(L^{137})
-0.18 (L^{137})		-5.36/-5.37(L^{136})		-4.63(L^{136})	-2.03/-2.03(S^{137})		-2.64(S^{137})	
-7.49 (H^{136})	-7.60/-7.60(H^{136})	-7.60/-7.60(H^{135})	-7.62(H^{136})	-7.62(H^{135})	-6.80/-6.08(H^{136})	-6.25/-6.25(H^{136})	-7.44(H^{136})	-7.44(H^{136})
-7.56 (O^{135})	-8.82/-8.82(O^{135})	-8.82/-8.82(O^{134})	-8.59(S^{135})		-7.46/-7.46(O^{135})	-7.46/-7.46(O^{135})	-8.18(O^{135})	-7.89(O^{135})
	-9.07/-9.08(O^{134})	-9.07/-9.07(O^{133})						
	-9.33/-9.33(S^{133})							
				**dC:::dG^{oxo}**				
-0.17 (O^{136})						-0.14/-0.14(L^{135})		-0.13(L^{135})
-0.45 (L^{135})		-4.92/-4.93(L^{134})		-4.15(L^{134})	-2.66/-2.66(S^{135})		-3.75(H^{135})	
-7.07 (H^{134})	-8.29/-8.29(H^{134})	-8.29/-8.29(H^{133})	-8.23(S^{134})		-6.96/-6.96(H^{134})	-6.75/-6.75(H^{134})	-6.94(S^{134})	-6.94(H^{134})
-8.20 (O^{133})	-8.95/-8.92(S^{133})		-8.40(H^{133})	-8.38(H^{133})	-7.30/-7.30 (O^{133})	-6.98/-6.98(O^{133})		
				**dC:::dG**				
-0.15(O^{132})						-0.12/-0.12(L^{131})		-0.11(L^{131})
-0.42 (L^{131})		-5.05/-5.05(L^{130})		-4.21(L^{130})	-2.64/-2.64(S^{131})		-3.72(H^{131})	
-7.18 (H^{130})	-8.25/-8.28(H^{130})	-8.27/-8.28(H^{129})	-8.27(H^{130})		-7.06/7.06(H^{130})	-6.73/-6.73(H^{130})	-7.06(S^{130})	-7.06 (H^{130})
-8.18 (O^{129})	-9.03/-9.03(S^{129})		-8.40(S^{129})	-8.75(H^{129})	-7.28/-7.28(H^{129})	-7.09/7.09(H^{129})	-7.69(S^{129})	-7.02(H^{129})
	-9.04/-9.04(O^{128})	-9.03/-9.03(O^{128})	-9.18(O^{128})	-9.18(O^{128})				
